# The photoinduced β-carotene synthesis in *Blakeslea trispora* is dependent on WC-2A

**DOI:** 10.3389/fmicb.2025.1554367

**Published:** 2025-03-25

**Authors:** Qiang Zheng, Kaili Zhu, Ke Wang, Yi Wang, Xiaobin Yu, Wei Luo

**Affiliations:** ^1^Modern Industrial College of Traditional Chinese Medicine and Health, Lishui University, Lishui, China; ^2^The Key Laboratory of Carbohydrate Chemistry and Biotechnology, Ministry of Education, School of Biotechnology, Jiangnan University, Wuxi, China; ^3^Department of Biological and Agricultural Engineering, University of California, Davis, Davis, CA, United States

**Keywords:** *Blakeslea trispora*, photoreceptor, β-carotene, GST pull-down, RNA interference

## Abstract

β-Carotene, a high value-added natural pigment, is currently produced industrially in *Blakeslea trispora*. Although photoinduced carotenoid synthesis has been identified in some filamentous fungi, there are still relatively few studies focusing on *B. trispora* and its potential mechanisms. In this study, an integrated strategy—including correlation analysis of gene expression, bioinformatics analysis, protein interaction, and RNA interference—was adopted to elucidate photoinduced β-carotene synthesis in *B. trispora*. Light wavelength, intensity, and irradiation duration stimulated the transcription of photoreceptors [*btwc-1* (*a, b, c*) and *btwc-2* (*a, b, c, d*)] and carotenoid structural genes (*carB* and *carRA*). The transcription of photoreceptor genes showed significant or high correlation with carotenoid structural genes under continuous or short-term, high-intensity blue light irradiation. To elucidate the role of photoreceptors in carotenoid synthesis, the interaction between BTWC-1 and BTWC-2 was predicted. Furthermore, Glutathione S-Transferase (GST) pull-down assays showed that only BTWC-1C and BTWC-2A could interact to form complexes. Inhibition of *btwc-2a* expression under dark conditions did not affect β-carotene accumulation or the transcription of *carB* and *carRA*, but did reduce these parameters under blue light irradiation, indicating that *btwc-2a* mediates photoinduced β-carotene synthesis in *B. trispora*.

## Introduction

The biosynthesis of light-induced carotenoids is widespread in filamentous fungi, including *B. trispora, Mucor circinelloides*, and *Phycomyces blakesleeanus*. Multiple studies have shown that the blue-light receptor regulates the expression levels of carotenoid structural genes *carB* (encoding lycopene dehydrogenase) and *carRA* (encoding lycopene synthase) by forming the White Collar Complex (WCC) and binding to photo-responsive promoter elements, thereby promoting β-carotene accumulation (Arrach et al., 2001; Velayos et al., 2000; Ruiz-Hidalgo et al., 1997).

The genes *al-1* and *al-2* (homologous to *carB* and *carRA*), as well as the geranylgeranyl pyrophosphate (GGPP) synthase gene *al-3* in *Neurospora crassa*, are all induced by light (Schmidhauser et al., 1990), with *al-3* expression depending on the light induction of *al-1* and *al-2* (Bieszke et al., 2007). Blue light stimulation in *N. crassa* leads to the production of carotenoids mediated by WCC. After light exposure, WCC transiently binds to the promoters of *al-3* and other light-induced genes, and acetylation of histone H3 associated with the LRR promoter element (containing the GATA-X15-GATA core sequence) regulates carotenoid synthesis (Grimaldi et al., 2006). Carotenoid biosynthesis in *B. trispora* is induced by various environmental factors (blue light, sexual interaction, retinol, and aromatic compounds), related to a photoreceptor with a flavin chromophore (Luo et al., 2020b; Corrochano and Garre, 2010). By extracting proteins from wild-type *B. trispora*, specific slow complexes were shown to form upon binding to DNA motifs in intergenic regions, correlating well with the kinetics of *carB* and *carRA* transcript accumulation; subsequent studies proved this complex to be the MAD complex (homologous to the WCC complex; Sanz et al., 2010). Carotenoid synthesis is also induced by blue light and mediated by photoreceptors in *P. blakesleeanus* (Cerdá-Olmedo, 2001) and *M. circinelloides* (Nicolas et al., 2009). Three functionally differentiated photoreceptor genes (*mcwc-1a, mcwc-1b, mcwc-1c*) have been identified (Silva et al., 2006, 2008), where *mcwc-1c* mainly regulates carotenoid production in response to blue light, *mcwc-1a* primarily controls phototropism, and *mcwc-1b* links carotenoid synthesis with development (Navarro et al., 2013). In *Sordaria fimicola, sfwc-1* (homologous to *wc-1*) mediates light-induced carotenoid biosynthesis, with carotenoid production reduced under light conditions when the *sfwc-1* LOV domain is knocked out (Krobanan et al., 2019). Similarly, in *Fusarium asiaticum*, knocking out *fawc-1* (homologous to *wc-1*) or *fawc-2* (homologous to *wc-2*) leads to decreased carotenoid accumulation (Tang et al., 2020).

In *B. trispora*, light stimulation can induce the expression of carotenoid structural genes and affect β-carotene synthesis (Luo et al., 2021). Three blue-light receptors WC-1 (A, B, and C) in *B. trispora* have been identified, which contain flavin binding domains to reversibly bind chromophore groups (FAD and FMN); WC-1A determines the phototropism of mycelium, while WC-1C is related to light-induced carotenogenesis (Luo et al., 2020b). Interestingly, WC-2 proteins in *B. trispora* also have multiple homologs (2A, 2B, 2C, and 2D), which have been identified in recent year (Ge et al., 2021). Since *B. trispora* is an industrially relevant strain for β-carotene production but lacks detailed research on light-induced β-carotene synthesis and its molecular mechanisms, the role of photoreceptor-mediated β-carotene synthesis in *B. trispora* remains incompletely understood, hindering development of its photosynthetic biology. Therefore, this study aimed to clone and express the blue-light receptors of *B. trispora* and to clarify their roles in β-carotene biosynthesis, providing a theoretical basis for the regulatory mechanism of photoreceptor-mediated β-carotene synthesis in *B. trispora*.

## Materials and methods

### Strain and main reagents

*B. trispora* NRRL2896 (–) was obtained from the laboratory collection, and *Agrobacterium tumefaciens* LBA4404 was purchased from Beijing Coolaber Company. The RNA extraction kit and RNA reverse transcription cDNA kit were purchased from Yisheng Biotechnology Co., Ltd. Western blot-related reagents such as Western fast transfer solution, biotin-labeled goat anti-rabbit IgG (H+L), and biotin-labeled goat anti-mouse IgG (H+L) were purchased from Shanghai Biyun Tian Biotechnology Co., Ltd. Primers were chemically synthesized by Suzhou Genewiz Biotechnology Co., Ltd.

### Cultivation methods

The spore suspension of *B. trispora* preserved in 20% glycerol was spread on PDA solid medium using a disposable spreader, and then cultured at 25°C for 4–5 days, during which the mycelium spread across the plate and formed numerous spores. Sterilized 20% glycerol was added to the medium, and spores were collected, diluted to 1.0 × 10^6^ spores·mL^−1^ using a hemocytometer, and spread on PDA medium. The plates were incubated in a light incubator at 25°C with protection from light. After 3 days of dark cultivation, the PDA plates were inverted so that the *B. trispora* mycelium could be exposed to different light conditions. The light source and intensity were adjusted in the incubator, with blue light (wavelength 400–500 nm) and red light (wavelength 620–720 nm) as LED monochromatic lights, and white light as LED mixed light. During dark incubation, plates were wrapped in aluminum foil. Mycelia were collected under red light for subsequent analyses of transcriptional levels and β-carotene content.

### Quantitative real-time PCR

Before RNA extraction, experimental equipment was treated with a solid-phase purifying agent to remove RNase and prevent RNA degradation. The *B. trispora* mycelia were placed in a pre-cooled mortar with liquid nitrogen and ground thoroughly until turning into a white powder. Total RNA was extracted according to the instructions of the column fungal total RNA extraction and purification kit. The purity and concentration of the extracted RNA were measured with a nucleic acid quantifier, and agarose gel electrophoresis was performed to check for possible degradation. First-strand cDNA was synthesized from RNA using the Hifair^®^ II 1st Strand cDNA Synthesis SuperMix for qPCR. Primers were designed according to standard real-time fluorescence quantitative PCR principles. Using Hieff^®^ qPCR SYBR Green Master Mix (High Rox Plus), the 20 μL reaction system contained 10 μL UltraSYBR mix, 2.8 μL cDNA (50 ng·μL^−1^), 0.4 μL of both upstream and downstream primers (μmol·L^−1^), and 6.4 μL ddH_2_O. The reaction conditions were 95°C for 10 min, followed by 40 cycles of 95°C for 15 s and 60°C for 30 s. The cDNA template was diluted to an appropriate concentration, and each group had three replicates (Schmidt et al., 2005). The *tef1* gene was selected as the housekeeping gene due to its stable expression in *Trichoderma* sp. and gene transcription levels were calculated using the 2^−ΔΔCt^ method for relative quantification (Luo et al., 2020a).

### Bioinformatics analyses

The basic physicochemical properties, structure, and functions of the three BTWC-1 and four BTWC-2 blue-light receptor proteins were predicted using online tools. The multimer model in AlphaFold v2.3.1 was employed for structural prediction (Chu et al., 2023), while ZDOCK v3.0.2 was used for protein–protein multimer structure prediction (Yang et al., 2021). The resulting multimeric structures were analyzed with Pymol v2.5.0 to identify amino acid pairs potentially forming hydrogen bonds (Xue et al., 2016). The binding affinity and dissociation constants of predicted multimers were obtained using PRODIGY v2 (Kastritis et al., 2014). The remaining bioinformatics methods and websites are listed in Supplementary Table S1.

### Prokaryotic expression, purification and *in vitro* interaction analysis of BTWC-1 and BTWC-2

The prokaryotic expression vectors for BTWC-1 were constructed previously in our laboratory, carrying His tags on both ends for protein purification and Western blot detection. In this study, the prokaryotic expression vector for BTWC-2 was constructed, in which each BTWC-2 target protein was fused with a GST tag for GST magnetic bead purification and subsequent GST pull-down experiments. Using *B. trispora* cDNA as a template, the target genes *btwc-2a, btwc-2b, btwc-2c*, and *btwc-2d* were individually amplified, and the pET-28a(+) vector was linearized by reverse PCR. The GST tag was amplified from a laboratory-preserved pET-28a-GST-wc-2 template, and connected to the target gene via homologous arms. The amplified products were checked by electrophoresis and purified using a rapid DNA purification kit. After calculating fragment concentrations, the vector and inserts were subjected to homologous recombination and transformed into *E. coli* BL21(DE3) or Rosetta(DE3) competent cells. Recombinant protein expression strains of BTWC-1 (A, B, C) and BTWC-2 (A, B, C, D) stored in glycerol were inoculated in 5 mL LB medium and cultured at 37°C for 12–16 h. Cultures were transferred to fresh LB medium at 37°C until OD_600_ reached 0.6–0.8. Protein expression was then induced with 0.4 mM IPTG at 16°C for 20 h for pColdII-*btwc-1a* (*E. coli* BL21; Luo et al., 2020b), pET-28a-*btwc-1b*-His (*E. coli* BL21; Liang et al., 2025), pColdII-*btwc-1c* (*E. coli* BL21); or with 0.3 mM IPTG at 30°C for 6–8 h for pET-28a-*btwc-2a* (*E. coli* BL21), pET-28a-*btwc-2b* (*E. coli* BL21), and pET-28a-*btwc-2d* (*E. coli* Rosetta). After induction, cells were harvested by centrifugation at 4°C, 8,000 rpm for 5 min, washed with PBS three times, disrupted by sonication, and the supernatant was collected as the crude protein solution. Protein expression was evaluated by 12% SDS-PAGE. His-tagged proteins were purified using His tag purification columns; GST-tagged proteins were purified using GST magnetic beads.

The purified GST-tagged bait proteins GST-BTWC-2 (A, B, C, D) and the His-tagged target proteins His-BTWC-1 (A, B, C) were then used for GST pull-down assays. The eluted products were assessed by Western blot. The multimer model in Alphafold v2.3.1 was used for flexible docking predictions; the binding affinity and dissociation constant between BTWC-1C and BTWC-2A were evaluated with PRODIGY v2.0; Pymol v2.5.0 was employed for structural analysis and identification of interacting amino acid pairs. The predicted binding sites were compared with the identified structural domains.

### RNA interference

By PCR cloning and sequencing to verify the splicing pattern, the cDNA sequence of *bwtc-2a* from *B. trispora* was obtained as the template for double-stranded RNA (dsRNA). Online tools DSIR (http://biodev.cea.fr/DSIR.html) and GenScript (https://www.genscript.com/tools/sirna-target-finder) were used to design siRNA sequences, and the resulting candidates were compared with the JGI database to exclude sequences homologous to other genes. Two *bwtc-2a* siRNA sequences were selected. Given that their GC and AT contents are the same, a shuffled shRNA sequence was chosen as the negative control (Supplementary Table S2).

A mouse mU6 shRNA promoter—already demonstrated to function in *B. trispora*—was used here (Yang et al., 2024). The mU6 shRNA promoter fragment was amplified by circular PCR from the plasmid pmU6-gRNA, and the linear vector fragment was obtained by circular PCR from plasmid pCambia1303. The downstream primer of the mU6 shRNA promoter fragment and the upstream primer of the linearized pCambia1303 carried homologous arms for shRNA formation, while the upstream primers of pCambia1303 carried homologous arms for the shRNA. The target vector was constructed by seamless cloning and introduced into *E. coli* DH5α; then, *A. tumefaciens* invaded the protoplasts of *B. trispora* to transform the vector.

### Correlation and significance analyses

Spearman's correlation analyses via SPSSPRO online tool were used to obtain correlations of the relative transcription levels between photoreceptors (*btwc-1* and *btwc-2*) and carotenoid structural genes (*carB* and *carRA*). The Spearman correlation coefficient is suitable for non-normally distributed data. A two-tailed test was first performed to determine whether a statistically significant relationship existed. If significant, correlation was assumed; if not, no correlation was found. Finally, the positive and negative correlation coefficients and the degree of correlation were analyzed. *R* is the correlation coefficient; | *R* |=1 indicates completely correlated; 0.8 < | *r* | < 1 indicates highly correlated; 0.5 < | *r* | ≤ 0.8 indicates significantly correlated; 0.3 < | *r* | ≤ 0.5 denotes low correlated; 0 < | *r* | ≤ 0.3 denotes weakly correlated; and | *r* |=0 denotes zero correlated. The statistical analysis data were taken from the mean ± standard deviation of three parallel samples. Differences in experimental groups were assessed by one-way ANOVA (SPSSPRO). 0.01 < *p* < 0.05, denotes a significant difference, while *p* < 0.01 represents a very significant difference. Cohen's *d* value indicates effect size, with 0.20, 0.50, and 0.80 considered the thresholds for small, medium, and large differences, respectively.

### Determination of β-carotene accumulation

Mycelia at different culture times were collected under red light, placed in a 10 mL centrifuge tube wrapped in foil, and washed thrice with distilled water. The washed mycelia were dried at 45°C for 48 h under vacuum, and the dry weight (DW) was measured. The mycelia were then ground into powder with quartz sand and repeatedly extracted with anhydrous ethanol until colorless. The extracts were pooled, supplemented with 2% BHT, and centrifuged (4°C, 8,000 rpm, 2 min) for β-carotene quantification by HPLC using a Zorbax SB-C18 column. Acetonitrile (15%, v/v) served as the mobile phase at 1.0 mL/min, with detection at 450 nm.

## Results and discussion

### Effects of irradiation conditions on the expression of photoreceptors and carotenoid structural genes

**Figure 1:**
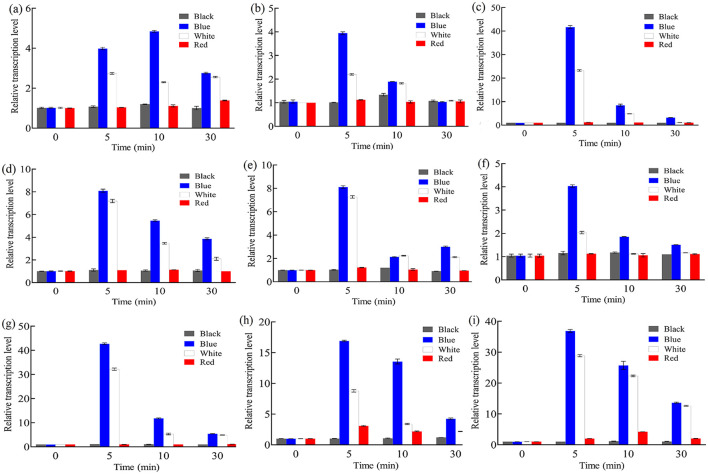
Relative transcription levels of *btwc-1, btwc-2*, and carotene structural genes under irradiation of different wavelength light at different times. **(A)** *btwc*-*1a*. **(B)** *btwc*-*1b*. **(C)** *btwc*-*1c*. **(D)** *btwc*-*2a*. **(E)** *btwc*-*2b*. **(F)** *btwc*-*2c*. **(G)** *btwc*-*2d*. **(H)** *carB*. **(I)** *carRA*. Black, blue, white, and red denote that strains were cultured in the darkness, blue light, white light, and red light, respectively. All strains were pre-cultured in the darkness for 3 days, at which time the transcription levels of these genes were used as controls. Three biological replicates were conducted for each experiment.

Since light induces carotenoid synthesis in *B. trispora*, expression of photoreceptors should mediate carotenogenesis via carotenoid structural genes (*carRA* and *carB*). To clarify the relationship between them, we investigated the transcription levels of photoreceptors and carotenoid structural genes under different light irradiation conditions. After 3 days of dark cultivation, total RNA was extracted from *B. trispora* mycelia, reverse-transcribed into cDNA, and analyzed by real-time PCR for transcription levels at different time points under continuous darkness, blue light, white light, and red light. As shown in Figure 1, *btwc-1 (a, b, c), btwc-2 (a, b, c, d), carRA*, and *carB* maintained relatively low expression under continuous darkness or red light, while transcription of these genes rapidly increased within 0–10 min under both blue and white light. These results suggest that blue light irradiation has a greater impact on gene transcription than white light, whereas red light has no effect.

**Figure 2:**
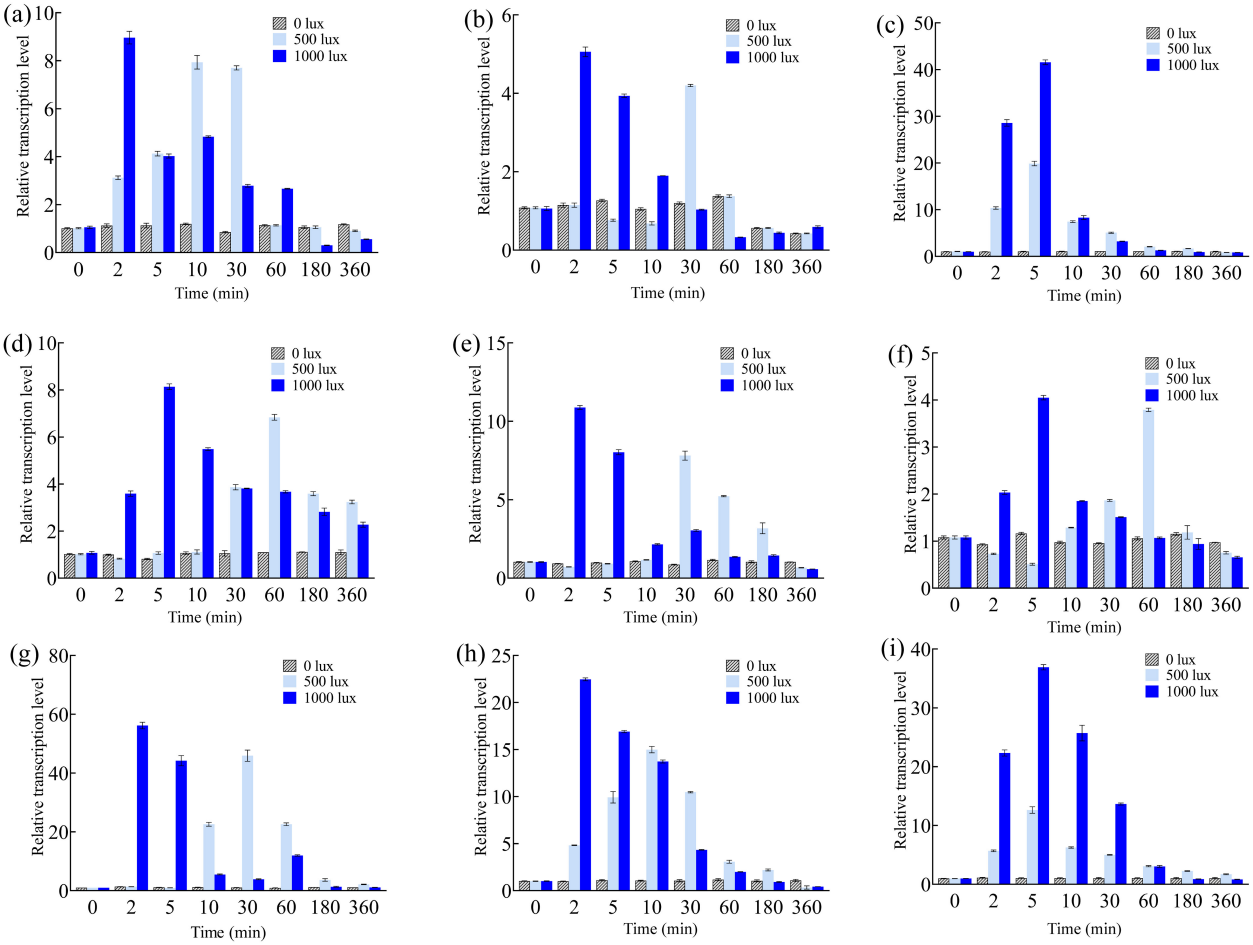
Relative transcription levels of *btwc-1, btwc-2*, and carotene structural genes under blue light irradiation of different intensities at different times. **(A)** *btwc*-*1a*. **(B)** *btwc*-*1b*. **(C)** *btwc*-*1c*. **(D)** *btwc*-*2a*. **(E)** *btwc*-*2b*. **(F)** *btwc*-*2c*. **(G)** *btwc*-*2d*. **(H)** *carB*. **(I)** *carRA*. All strains were pre-cultured in the dark for 3 days, at which time the transcription levels of these genes were used as controls. Three biological replicates were conducted for each experiment.

Next, mycelia were irradiated with different intensities of blue light for various durations, and the relative transcription levels were measured. As shown in Figures 2A–G, *btwc-1a* and *btwc-1b* reached their highest expression at 2 min under high intensity (1,000 lux) but at 10–30 min under low intensity (500 lux). The response speed of *btwc-1c* to light was intensity-independent, with transcription peaking at 5 minutes; however, its expression under low intensity was significantly lower than under high intensity. For *btwc-2*, the time to maximum transcription was delayed from ~2–5 min under high intensity to ~30–60 min under low intensity. The relative transcription levels of *btwc-1a, btwc-1b, btwc-1c, btwc-2a, btwc-2b, btwc-2c*, and *btwc-2d* all peaked at 2–5 min under high-intensity irradiation, at 9.1-, 5.1-, 41.9-, 8.1-, 8.0-, 4.0-, and 56.2-fold higher than in the darkness, respectively. As light exposure continued, these transcription levels decreased. Except for *btwc-2a*, whose transcription remained higher than that in the darkness even at 180–360 min, the other six genes eventually decreased below darkness levels. Therefore, the seven photoreceptor genes showed no significant changes under continuous darkness but displayed transient increases after blue light irradiation, indicating a rapid response to high-intensity light.

**Figure 3:**
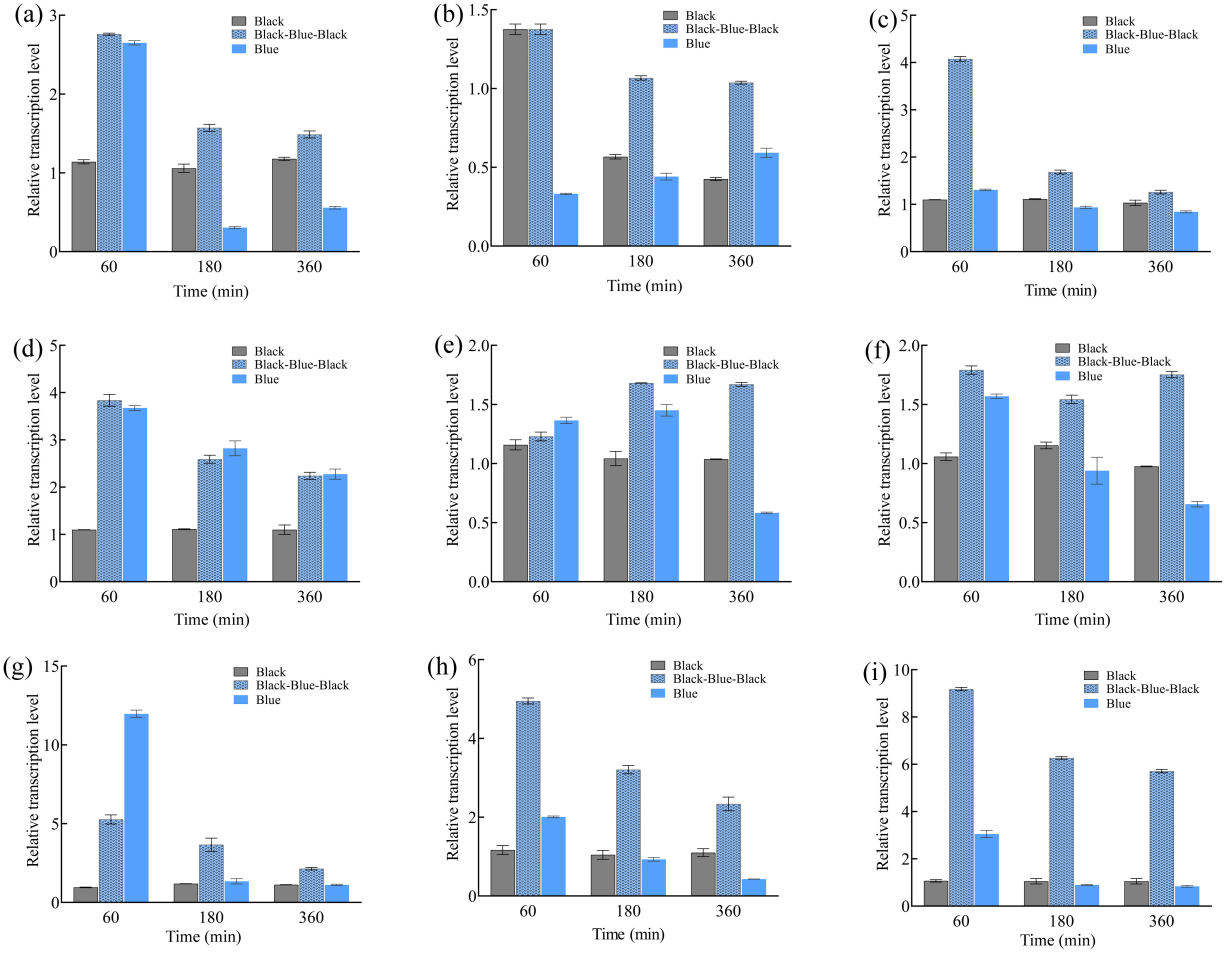
Relative transcription levels of *btwc-1, btwc-2*, and carotene structural genes under continuous (360 min) or short-term (30 min) blue light (1,000 lux) irradiation at different times. **(A)** *btwc*-*1a*. **(B)** *btwc*-*1b*. **(C)** *btwc*-*1c*. **(D)** *btwc*-*2a*. **(E)** *btwc*-*2b*. **(F)** *btwc*-*2c*. **(G)** *btwc*-*2d*. **(H)** *carB*. **(I)** *carRA*. Black and blue denote that strains were cultured in the darkness and blue light, respectively. Black-blue-black represents strains were exposed to blue light (1,000 lux) for 30 min and then moved into darkness. All strains were pre-cultured in the darkness for 3 days, at which time the transcription levels of these genes were used as controls. Three biological replicates were conducted for each experiment.

Similarly, *carB* expression peaked at 2 min under high-intensity irradiation (22.3-fold higher than in the darkness; Figure 2H). Under low intensity, *carB* peaked at 10 min (14.9-fold). The *carRA* expression pattern under different intensities was similar, peaking at 5 min under both intensities but reaching 36.8- and 12.8-fold above darkness at low and high intensity, respectively. Additionally, short-term (30 min) blue light irradiation was performed to investigate transcription of the nine genes (Figure 3). Their expression levels trended downward over time, yet they remained higher than in the darkness. Under continuous blue light, the gene transcription eventually dropped below darkness levels with prolonged exposure.

### Transcription correlation analyses

These results show that the transcription of *btwc-1, btwc-2, carB*, and *carRA* is induced by blue light, with the nine genes exhibiting distinct transcriptional patterns under different irradiation conditions. After initial stimulation, gene transcription increased and then decreased. To investigate the intrinsic relationship between gene expression and light stimulation, relative transcription levels of *btwc-1, btwc-2*, and *carB, carRA* were correlated (Table 1). Under continuous blue light (500 lux), *btwc-1a* (or *btwc-1c*) was highly correlated with *carB* and *carRA*. Under both continuous (1,000 lux) and short-term (1,000 lux) blue light, correlations were found between most photoreceptors (except *btwc-2c*) and *carB, carRA*. In particular, *btwc-1a, btwc-1c*, and *btwc-2a* showed high correlation with *carB* and *carRA*. Moreover, correlation coefficients under short-term irradiation generally equaled or exceeded those from continuous irradiation.

**Table 1:** Correlation analysis of relative transcriptional levels between *btwc-1, btwc-2* and *carB, carRA*.

**Gene**	**Continuous blue light irradiation (500 lux)**		**Continuous blue light irradiation (1,000 lux)**		**Short-term blue light irradiation (1,000 lux)**	
	***carB***	***carRA***	***carB***	***carRA***	***carB***	***carRA***
*btwc-1a*	0.958^***^	0.857^***^	0.952^***^	0.881^***^	0.952^***^	0.905^***^
*btwc-1b*	0.405	0.19	0.786^**^	0.709^**^	0.833^**^	0.714^**^
*btwc-1c*	0.81^**^	0.952^***^	0.966^***^	0.952^***^	0.976^***^	0.976^***^
*btwc-2a*	0.024	−0.167	0.81^**^	0.857^***^	0.905^***^	0.929^***^
*btwc-2b*	0.405	0.024	0.705^**^	0.81^**^	0.738^**^	0.81^**^
*btwc-2c*	0.381	−0.095	0.524	0.452	0.5	0.286
*btwc-2d*	0.429	0.048	0.857^***^	0.762^**^	0.926^***^	0.833^**^

^***^p < 0.01 and ^**^0.01 < p < 0.05; the values in the table are the correlation coefficient.

The differences in transcription timing and amplitude among these photoreceptors indicate functional diversification of multiple *wc* genes in *B. trispora*. The light-induced transcription of *btwc-1* and *btwc-2* is transient; under continuous illumination, transcription eventually declines, possibly reflecting “photoadaptation” (Avalos et al., 2017). Under photoadaptation, light-induced genes are transiently activated, and mRNA levels decrease to a stable baseline upon prolonged irradiation. Even photoadaptation-determining gene (*vvd*) has been identified in *N. crassa*, no homologous of *vvd* have been clarified in *B. trispora*, suggesting that another mechanism for photoadaptation is operating (Schmoll et al., 2010). Under varying intensities, the transient expression of *btwc-1* and *btwc-2* overlaps with β-carotene biosynthetic gene transcription. This is consistent with early light response in *N. crassa*, where binding to light response elements is transient, and target gene transcription occurs concurrently (Dasgupta et al., 2015). Previous studies suggest that different light intensities can promote β-carotene accumulation to varying degrees. Lower light intensities generally yield lower β-carotene accumulation, whereas intensities beyond a threshold do not further enhance β-carotene (Luo et al., 2021). Here, *carB* and *carRA* are transcriptionally activated by a certain light intensity threshold (Figure 2). Additionally, many studies have used continuous irradiation, but recent findings indicate that non-continuous exposure can significantly boost β-carotene accumulation. Short-term blue light avoids “photoadaptation,” possibly explaining its efficiency in promoting β-carotene (Luo et al., 2021). The strong correlation between photoreceptor gene transcription and carotenoid structural genes suggests that light-stimulated carotenoid synthesis may occur by regulating the transcription of carotenoid biosynthetic genes—an essential regulatory step (Rodriguez-Ortiz et al., 2013). In *N. crassa*, WC-1 and WC-2 form the WCC complex to trigger transient transcription of carotenoid structural genes after light activation of *wc-1* and *wc-2* (Castrillo et al., 2018).

### Expression, purification and *in vitro* interaction analyses of BTWC-1 and BTWC-2

BTWC-1A, BTWC-1C, BTWC-2A, BTWC-2C, and BTWC-2D each contain a zinc finger domain, whereas BTWC-1B and BTWC-2B do not (Supplementary Figure S1). Zinc finger domains are associated with gene expression regulation, for example, binding to cis-elements such as the I-box (Rose et al., 1999). Photoreceptors containing such domains can specifically bind to light-regulated promoter sequences, thus regulating downstream gene expression (Linden and Macino, 1997; Nicolas et al., 2008; Brenna and Talora, 2019). In the model organism *Phycomyces*, WC-1 and WC-2 form the WCC complex via the PAS domain and then regulate downstream targets (Linden and Macino, 1997). In *M. circinelloides*, the MAD complex (homologous to WCC) induces carotenoid synthesis by regulating the transcription of pathway genes (Corrochano and Garre, 2010; Sanz et al., 2010). In this study, three BTWC-1 and four BTWC-2 proteins in *B. trispora* were found to contain PAS domains, and cis-element analysis revealed multiple light-responsive elements (G-box, Sp1, TCCC-motif, DRE1, chs-CMA2a, I-box, TCT-motif, GATA-motif, and Box 4) in the *carB* and *carRA* promoters.

The dimerization structures of BTWC-1 and BTWC-2 proteins were predicted using ZDOCK v3.0.2 rigid docking and then the top 10 ranked multimeric structures were selected using a scoring function. The binding affinity and dissociation constants of the 12 groups of BTWC-1 and BTWC-2 dimers predicted using PRODIGY v2.0 are shown in Table 2. The dissociation constants that < 1e-10 indicate the possibility of strong binding between all 12 groups of proteins (Vangone and Bonvin, 2015), with the strongest interactions predicted for BTWC-1A/BTWC-2A, BTWC-1C/BTWC-2A, and BTWC-1C/BTWC-2D.

**Table 2:** Prediction of binding affinity and dissociation constant of BTWC-1 and BTWC-2 dimers.

**Dimerization**	**Combining affinity (kcal.mol^−1^)**	**Dissociation constant 25°C (M)**
BTWC-1A & BTWC-2A	−22.1	6.5e-17
BTWC-1A & BTWC-2B	−15.8	2.7e-12
BTWC-1A & BTWC-2C	−16.1	1.6e-12
BTWC-1A & BTWC-2D	−15.8	2.6e-12
BTWC-1B & BTWC-2A	−15.5	4.1e-12
BTWC-1B & BTWC-2B	−11.9	1.8e-09
BTWC-1B & BTWC-2C	−19.5	4.6e-15
BTWC-1B & BTWC-2D	−18.8	1.5e-14
BTWC-1C & BTWC-2A	−21.2	3.0e-16
BTWC-1C & BTWC-2B	−19.8	3.0e-15
BTWC-1C & BTWC-2C	−17.5	1.4e-13
BTWC-1C & BTWC-2D	−20.0	2.1e-15

**Figure 4:**
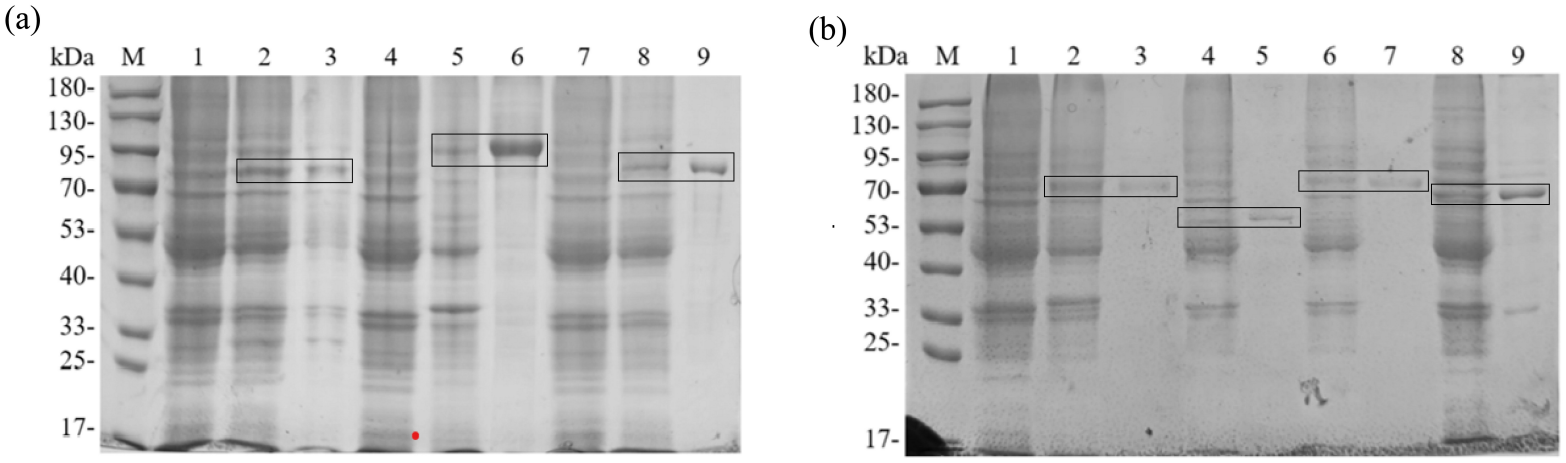
SDS-PAGE profiling of crude and purified BTWC-1 and BTWC-2 proteins (in square frames). M is Maker in Figures; **(A)**, lanes 1–9 are: pColdII control, BTWC-1A crude protein, BTWC-1A purified protein, pET-28a control, BTWC-1B crude protein, BTWC-1B purified protein, pColdII control, BTWC-1C crude protein, BTWC-1C purified protein, respectively. **(B)**, lanes 1–9 are: pET-28a control, BTWC-2A crude protein, BTWC-2A purified protein, BTWC-2B crude protein, BTWC-2B purified protein, BTWC-2C crude protein, BTWC-2C purified protein, BTWC-2D crude protein, BTWC-2D purified protein, respectively.

According to SMS2, the predicted molecular weights of the fusion-tagged BTWC-1A, BTWC-1B, BTWC-1C, BTWC-2A, BTWC-2B, BTWC-2C, and BTWC-2D proteins are 73, 88, 75, 69, 56, 70, and 66 kDa, respectively. As shown in Figure 4, the SDS-PAGE bands match these predictions, demonstrating suitability for subsequent GST pull-down.

**Figure 5:**
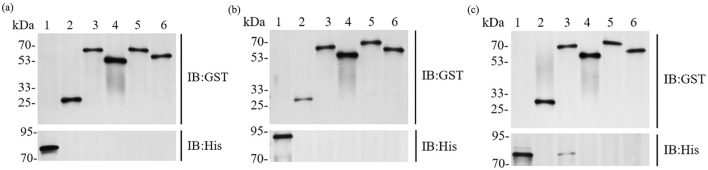
GST Pull down assay of the interaction between 12 sets of BTWC-1 and BTWC-2. In **(A)**, lanes 1–6 are BTWC-1A target protein positive control, GST negative control, HIS-BTWC-1Aeluted bands after co-incubation with GST-BTWC-2A, GST-BTWC-2B, GST-BTWC-2C, and GST-BTWC-2D, respectively; in **(B)**, lanes 1–6 are the positive control for BTWC-1B target protein, the positive control for HIS-BTWC-1B with GST-BTWC-2A, GST-BTWC-2B, GST BTWC-2A, GST-BTWC-2B, GST-BTWC-2B, GST-BTWC-2D, respectively; in **(C)**, lanes 1–6 are the positive control for BTWC-1C target protein, the GST negative control, the elution bands after co-incubation of HIS-BTWC-1C with GST-BTWC-2A, GST-BTWC-2B, GST-BTWC-2C, and GST-BTWC-2D, respectively.

GST pull-down experiments were conducted to confirm interactions between BTWC-1 and BTWC-2. GST-fused bait proteins GST-BTWC-2A, GST-BTWC-2B, GST-BTWC-2C, and GST-BTWC-2D bound to GST magnetic beads were co-incubated with His-BTWC-1A, His-BTWC-1B, or His-BTWC-1C. After washing, the complexes were eluted, separated by SDS-PAGE, and examined by Western blot with anti-GST and anti-His antibodies; the target proteins were positive controls, and GST plus target proteins served as negative controls. As shown in Figures 5A, B, no interaction was detected for His-BTWC-1A/1B with GST-BTWC-2A/2B/2C/2D. However, the co-incubation of His-BTWC-1C with GST-BTWC-2A yielded the same molecular weight band as the positive control for GST-BTWC-2A (Figure 5C), indicating that BTWC-1C and BTWC-2A bind *in vitro*.

Previous reports show that WC-1 and WC-2 jointly regulate carotenoid synthesis (Linden and Macino, 1997; Huang et al., 2022; Nelson et al., 1989), suggesting that BTWC-1C and BTWC-2A may co-regulate β-carotene in *B. trispora*. Further genetic manipulation is needed to confirm that BTWC-1C and BTWC-2A indeed mediate β-carotene production in *B. trispora*.

### Effect of *btwc-2a* on β-carotene synthesis

An RNA interference vector was constructed targeting *btwc-2a* to clarify the effects of *btwc-2a* on β-carotene synthesis in *B. trispora*. Total RNA was isolated at different time points, reverse-transcribed, and tested by real-time PCR to determine *btwc-2a* levels in the interfered and control strains. Two gene interfered strains were constructed to investigate whether there were significant differences in the interference efficiency.

**Figure 6:**
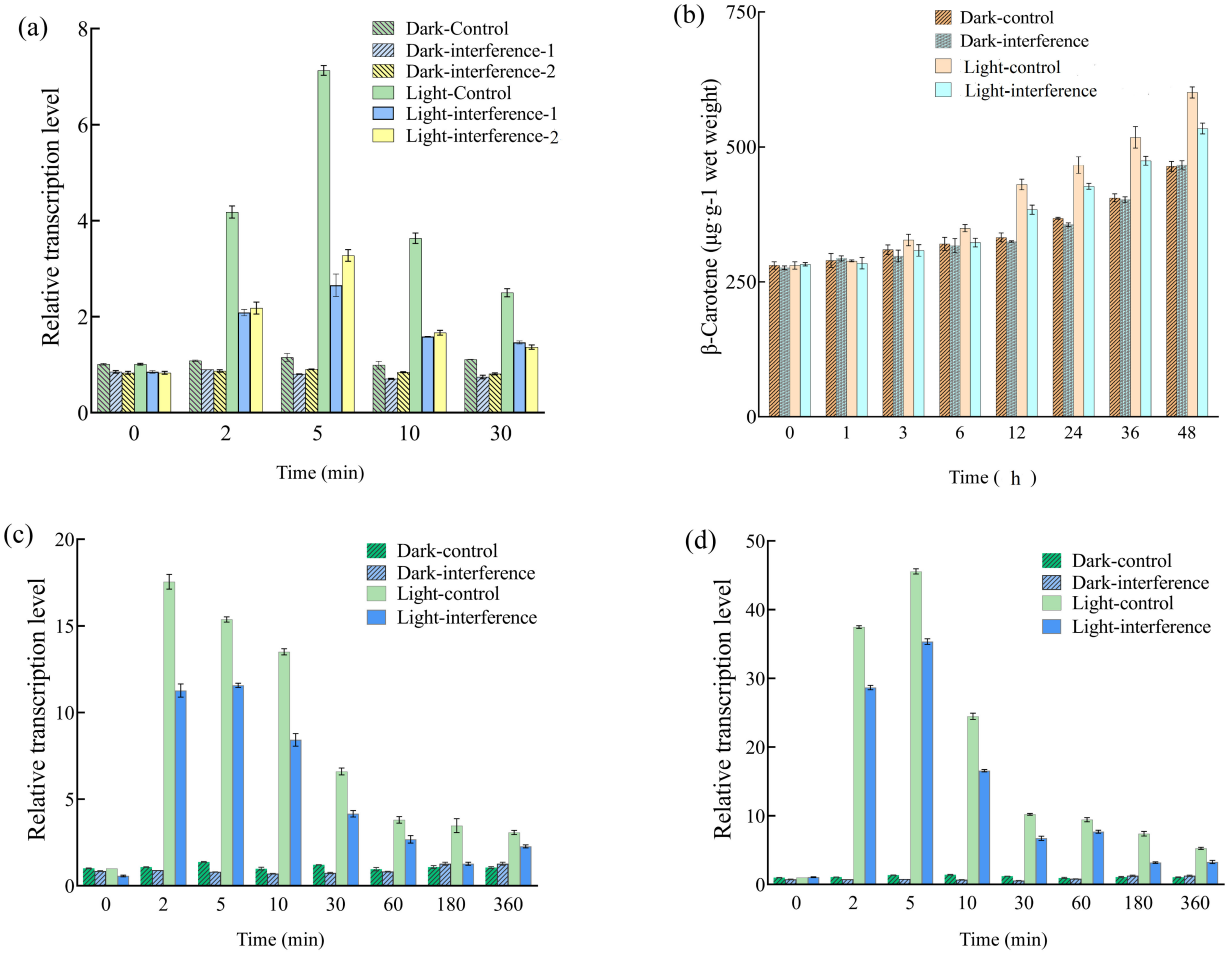
Relative transcription levels of *btwc-2a*, carotene structural genes, and β-carotene accumulation in RNA interfered and control strains. **(A)** Shows the relative transcription levels of *btwc-2a* in RNA interfered and control strains at different times. The control strain, interfered strain 1 (interfererence 1), and interfered strain 2 (interfererence 2) were *B. trispora* strains transformed with pCambia1303-mU6-RNAidz, pCambia1303-mU6-*btwc-2a*-RNAi1, and pCambia1303-mU6-*btwc-2a*-RNAi2, respectively; **(B)** shows the accumulation of β-carotene in *btwc-2a* interfered (strain 1) and control strains at different times under different light conditions; **(C, D)** show the relative transcription levels of *carB* and *carRA* in *btwc-2a* interfered (strain 1) and control strains at different times, respectively. Dark denotes that strains were cultured in the darkness, while light represents that strains were exposed to blue light (1,000 lux) for 30 min and then moved into darkness. All strains were pre-cultured in the darkness for 3 days, at which time the transcription levels of these genes were used as controls. Three biological replicates were conducted for each experiment.

As shown in Figure 6A, there were no significant differences (*p* > 0.05) in *btwc-2a* transcription levels between the two interfered strains, demonstrating the stability of this interference strategy. Meanwhile, *btwc-2a* transcription levels did not differ significantly between control and two interfered strains (*p* > 0.05) in the darkness, likely due to low *btwc-2a* expression in the dark. Under blue light, however, *btwc-2a* in both strains increased significantly relative to darkness, and the difference between the two strains was highly significant (*p* < 0.01), indicating that RNA interference targeting *btwc-2a* strongly inhibits its light-induced expression. Although *btwc-2a* expression in the interfered strain was still transiently activated, its peak expression (2–30 min) was consistently lower than in the control strain (*p* < 0.01).

In the subsequent study, strain 1 was chosen to determine the effect of interference applied to *btwc-2a* on β-carotene accumulation and transcription of carotenoid structural genes. As shown in Figure 6B, β-carotene accumulation in control and interfered strains under short-term blue light was ultimately 22.2% and 12.6% higher than in the darkness, respectively, indicating that short-term blue light promotes β-carotene accumulation. While there was no significant difference between the strains in the darkness, β-carotene levels in the interfered strain were 12.4% lower than in the control (*p* < 0.05) under blue light, suggesting that *btwc-2a* mediates light-induced regulation of β-carotene synthesis.

To investigate how *btwc-2a* interference reduced β-carotene, the transcription of *carB* and *carRA* was measured. As shown in Figures 6C, D, *carB* and *carRA* transcription in both control and interfered strains surged at 2–5 min before decreasing to stable levels under short-term blue light, and they displayed significant differences (*p* < 0.05). By contrast, *carB* and *carRA* remained at low levels with no significant change between 0 min and 360 min in the darkness. Considering the significantly lower β-carotene in interfered strains under short-term blue light (Figure 6B), it appears that β-carotene reduction is due to decreased *carB* and *carRA* expression upon *btwc-2a* RNA interference.

Our findings showed that *mcwc-1c* deficiency in *M. circinelloides* can be rescued by expressing *btwc-1c* demonstrating *btwc-1c* also determines β-carotene synthesis (Luo et al., 2020b). Additionally, our previous study indicated that interfering with *btwc-1c* significantly reduced transcription of structural genes and β-carotene production (Shen et al., 2022), while the present study displayed a much similar landscape for the effect of *btwc-2a* on β-carotene synthesis. The fact that BTWC-2A can bind BTWC-1C makes it reasonable to speculate that they mediated the synthesis of β-carotene through the formation of a complex.

## Conclusion

In this study, photoreceptors and carotenoid structural genes exhibit rapid light response to blue and white light, but do not respond to red light. In addition, high-intensity blue light irradiation can accelerate the transcription levels of photoreceptors and carotenoid structural genes to reach their highest values, while short-term blue light irradiation can maintain high transcription levels of these genes for a longer period. Correlation analysis showed that transcription of multiple photoreceptors was significantly correlated with that of carotenoid structural genes. The dimer binding affinity and GST pull-down analyses indicated that WC-1C was able to bind to WC-2A, and interfering with the expression of WC-2A under light irradiation inhibited the light induced synthesis of β-carotene.

These results demonstrate that the photoinduced β-carotene synthesis in *B. trispora* is mediated by photoreceptors. Our previous study indicates that WC-1C affects this process, while this study suggests that WCC complex formed by WC-2A with WC-1C to determine β-carotene synthesis. However, further studies are required to clarify how WCC complex mediate the synthesis of β-carotene.

## Data Availability

The original contributions presented in the study are included in the article/Supplementary material, further inquiries can be directed to the corresponding author.
