# Supplementary material for: The photoinduced β-carotene synthesis in Blakeslea trispora is dependent on WC-2A
Source: Front Microbiol. 2025 Mar 25;16:1554367. doi: 10.3389/fmicb.2025.1554367 (PMC11975959; doi:10.3389/fmicb.2025.1554367)
Supplement: Supplementary file 1 [file Data_Sheet_1.docx]

Table S1 Websites of bioinformatics analysis.

| Forecast projects | URL |
| --- | --- |
| Functional domains | SMART (https://smart.embl-heidelberg.de/help/) |
| Tertiary structure | Alphafold (https://alphafold.ebi.ac.uk/) |
| Subcellular localization | WoLF PSORT (https://wolfpsort.hgc.jp/) |
| Protein-protein interactions | STRING (https://www.string-db.org/) |
| Protein molecular weight | SMS2 (http://wwwtaibio.com/sms2/protein_mw.html) |

Table S2 Sequence of shRNA.

| Target genes | Target genes shRNA sequence (5ʹ-3ʹ) |
| --- | --- |
| *btwc-2a* (1) | GAAACTAGATGAAGATGAATTCAAGAGATTCATCTTCATCTA  GTTTCTTTTTT |
| *btwc-2a* (2) | GTAAGTCAATACAGAGAAATTCAAGAGATTTCTCTGTATTGA  CTTACTTTTTT |
| None (control) | TGATGGCGCATTAATATTATTCAAGAGATAATATTAATGCGCC  ATCATTTTTT |

Fig. S1 Prediction of functional domains of BTWC-1 and BTWC-2.
