# Supplementary figures and images for: The photoinduced β-carotene synthesis in Blakeslea trispora is dependent on WC-2A
Source: Front Microbiol. 2025 Mar 25;16:1554367. doi: 10.3389/fmicb.2025.1554367 (PMC11975959; doi:10.3389/fmicb.2025.1554367)

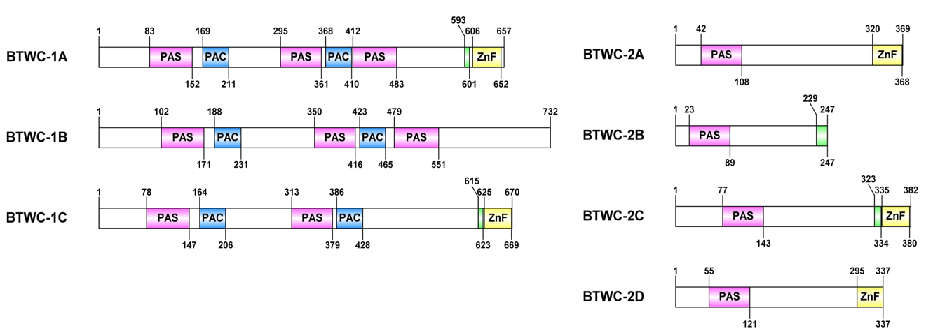

Supplement: Supplementary file 2 [file Image_1.tiff]
